# Efficient CO_2_ Reduction to Formate on CsPbI_3_ Nanocrystals Wrapped with Reduced Graphene Oxide

**DOI:** 10.1007/s40820-023-01132-3

**Published:** 2023-06-29

**Authors:** Minh Tam Hoang, Chen Han, Zhipeng Ma, Xin Mao, Yang Yang, Sepideh Sadat Madani, Paul Shaw, Yongchao Yang, Lingyi Peng, Cui Ying Toe, Jian Pan, Rose Amal, Aijun Du, Tuquabo Tesfamichael, Zhaojun Han, Hongxia Wang

**Affiliations:** 1 School of Chemistry and Physics, Faculty of Science, Queensland University of Technology, Brisbane, QLD 4001 Australia; 2 Centre for Materials Science, Queensland University of Technology, Brisbane, QLD 4001 Australia; 3 School of Chemical Engineering, The University of New South Wales, Kensington, NSW 2052 Australia; 4 Centre for Organic Photonics & Electronics (COPE), School of Chemistry and Molecular Biosciences, The University of Queensland, Brisbane, QLD 4072 Australia; 5 School of Engineering, The University of Newcastle, Callaghan, NSW 2038 Australia

**Keywords:** Perovskite nanocrystal, Electrocatalyst, Inorganic perovskite, CO_2_ reduction, Formate production

## Abstract

**Highlights:**

- A rational design of metal halide perovskites for achieving efficient CO_2_ reduction reaction was demonstrated.
- The stability of CsPbI_3_ perovskite nanocrystal (NCs) in aqueous electrolyte was improved by compositing with reduced graphene oxide (rGO).
- The CsPbI_3_/rGO catalyst exhibited > 92% Faradaic efficiency toward formate production with high current density which was associated with the synergistic effects between the CsPbI_3_ NCs and rGO.

**Abstract:**

Transformation of greenhouse gas (CO_2_) into valuable chemicals and fuels is a promising route to address the global issues of climate change and the energy crisis. Metal halide perovskite catalysts have shown their potential in promoting CO_2_ reduction reaction (CO_2_RR), however, their low phase stability has limited their application perspective. Herein, we present a reduced graphene oxide (rGO) wrapped CsPbI_3_ perovskite nanocrystal (NC) CO_2_RR catalyst (CsPbI_3_/rGO), demonstrating enhanced stability in the aqueous electrolyte. The CsPbI_3_/rGO catalyst exhibited > 92% Faradaic efficiency toward formate production at a CO_2_RR current density of ~ 12.7 mA cm^−2^. Comprehensive characterizations revealed the superior performance of the CsPbI_3_/rGO catalyst originated from the synergistic effects between the CsPbI_3_ NCs and rGO, i.e., rGO stabilized the α-CsPbI_3_ phase and tuned the charge distribution, thus lowered the energy barrier for the protonation process and the formation of *HCOO intermediate, which resulted in high CO_2_RR selectivity toward formate. This work shows a promising strategy to rationally design robust metal halide perovskites for achieving efficient CO_2_RR toward valuable fuels.
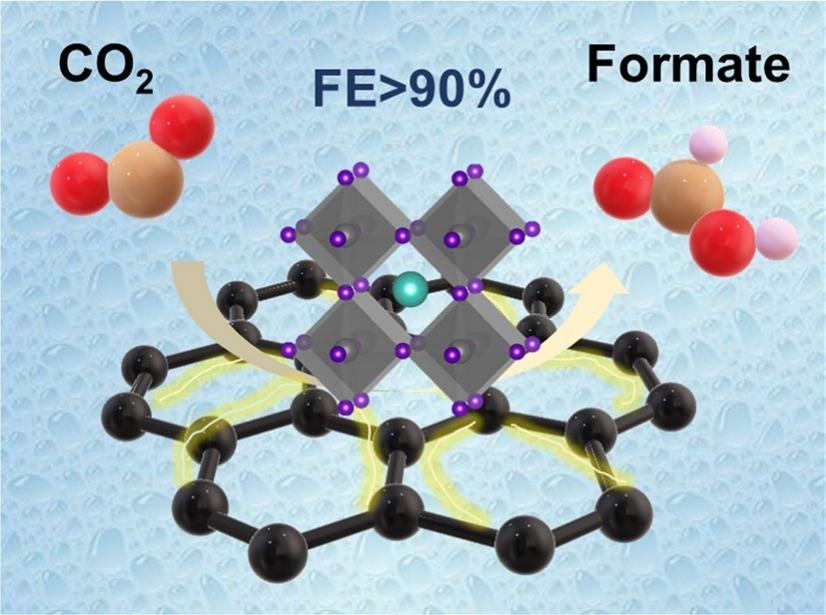

**Supplementary Information:**

The online version contains supplementary material available at 10.1007/s40820-023-01132-3.

## Introduction

Metal halide perovskites (MHPs) are recognized as one of the most effective materials for solar-to-electrical energy conversion [1–3]. Recently, the interesting electrical properties of MHPs have inspired the exploration of their applications in catalytic reactions including CO_2_ reduction, water splitting, organic synthesis, and dye degradation [4–12]. The versatility of chemical tailoring and ion combination of the MHPs makes them suitable for many different catalytic reactions [13–15]. However, the application of MHPs for electrocatalysis remains challenging due to their low stability, particularly under electrocatalytic conditions [16, 17].

The catalytic property of MHPs was firstly demonstrated in 2016 by Park et al. They used methylammonium lead triiodide (MAPbI_3_) MHP for solar-driven electrochemical hydrohalic acid splitting for H_2_ production, which achieved a Faradaic efficiency (FE) of almost 100% [18], where the MAPbI_3_ was stabilized by utilizing the precipitation–solubility equilibrium between the perovskite phase and the soluble ionic species in aqueous electrolyte. This opens an appealing chapter of metal halide perovskites for catalytic applications [19]. Especially, in the field of electrochemical reduction of CO_2_, the inorganic MHP CsPbBr_3_ exhibited significant electrocatalytic activity with FE of 32% for CH_4_ and 40% for CO [20]. In addition, mixed halides CsPb(Br_0.5_Cl_0.5_)_3_ MHP catalyst boosted solar-driven electrochemical production of CO from CO_2_ with a yield of 875 μmol g^−1^ and selectivity up to 99% [21], while the MAPbI_3_ catalyst incorporated in iron-porphyrin derived metal–organic frameworks delivered a high total yield of 1559 μmol g^−1^ for photocatalytic CO_2_ reduction to CO (34%) and CH_4_ (66%) [22]. These studies have shown the application potential of MHPs in electrochemical CO_2_RR toward valuable fuels. Although there are potential environmental and health risks associated with the use of lead-based MHP catalysts, research of this type of material for CO_2_RR is still important fundamentally. Pb-based catalysts such as Pb nanoparticles or Pt_n_Pb nanocrystals have shown excellent effectivity and selectivity in CO_2_RR [23–25], however the study of the potential of Pb-based metal halide perovskites for CO_2_ reduction is very limited. Moreover, from economical point of view, the much lower material cost and the ease of scalability of Pb-based MHP materials by solution method also make them very attractive compared to the precious metal-based catalysts for CO_2_RR, such as platinum which have been widely studied for CO_2_RR application.

Nevertheless, significant research challenges for the development of MHPs CO_2_RR catalysts remain [26]. One daunting issue hindering their application is the instability of MHPs in the majority of common CO_2_RR electrolytes. MHPs are highly unstable in polar solvents, especially under catalytic reaction conditions where radical species are involved. Hence, low-polarity solvents, such as ethyl acetate, acetonitrile, or toluene were previously used as electrolyte solvent to preserve the phase and optoelectrical properties of MHPs catalysts [4, 11, 27]. However, the catalytic performance of MHPs in these electrolytes is unfavourable due to the low solubility of CO_2_ in these low polarity solvents [28]. Therefore, catalyst design strategies based on hybrid MHPs with different materials, i.e., graphene oxide [29], Pt nanoparticles [30], metal–organic frameworks [31, 32], have been used to enhance the stability of the MHPs. For example, the hybrid CsPbBr_3_ nanocrystals (NCs) with graphitic carbon nitride showed enhanced stability in both acetonitrile/water and ethyl acetate/water solvent systems, while exhibited enhanced photocatalytic CO production rate of 149 µmol h^−1^ g^−1^ [27]. However, the lifetime of MHP-based catalyst is still far from the requirement for a substantial electrolysis system [33]. It is thus critical to resolve the instability of MHPs in the catalytic reaction conditions. In addition, most of the efforts have been devoted into the study of CsPbBr_3_ catalysts with the primary reduction products of CH_4_ and CO; whereas electrochemical CO_2_ reduction toward other valuable chemicals has not been demonstrated.

Herein, we demonstrate an inorganic CsPbI_3_ perovskite NCs wrapped with reduced graphene oxide (rGO) for CO_2_RR in aqueous electrolyte, which exhibited increased stability compared with the bare CsPbI_3_ perovskite NCs. In addition, the CsPbI_3_/rGO catalyst exhibited outstanding activity (*j*_*CO2RR*_ of 12.7 mA cm^−2^) and selectivity (FE_formate_ of 92%), which is attributed to the accumulated electron density near the active CsPbI_3_ perovskite structure induced by the rGO wrapping, as evidenced by experimental characterization and density functional theory (DFT) modelling. Together with the unravelled CO_2_RR mechanism of CsPbI_3_/rGO catalyst, this work unleashes the potential of inorganic MHPs as robust and efficient CO_2_RR catalysts for sustainable fuels production.

## Experimental Section

### Materials

Chemicals including Cs_2_CO_3_ (99.95%, Sigma Aldrich), Oleic Acid ($\ge$ 99%, Sigma Aldrich), Octadecene (Sigma Aldrich), PbI_2_ (99.99%, Sigma Aldrich), KHCO_3_, Oleylamine (Sigma Aldrich), reduced graphene oxide (Sigma Aldrich), Methyl acetate, Hexane was used as received without further purification.

### Synthesis of Perovskite NCs

The synthesis procedures were carried out following a published method in literature with some modifications [34]. Cs-Oleate was prepared by adding 202.8 mg of Cs_2_CO_3_ and 10 mL Octadecene (ODE) into a 100 mL three-neck flask. The flask was then degassed and dried under vacuum at 120 °C for 30 min. Subsequently, 0.63 mL of Oleic Acid (OA) was also added into the solution and the system was degassed for another 30 min. After that, the mixture was heated to 160 °C under N_2_ gas and stirred until all the Cs_2_CO_3_ was dissolved to make a clear solution. The Cs-oleate solution is kept at 100 °C under N_2_ gas for further use.

In another three-neck flask, 0.3 mg of PbI_2_ and 20 mL ODE were loaded. The flask also was degassed and dried under vacuum for 1 h at 120 °C. A mixture of 1.5 mL OA and 1.5 mL Oleylamine (OLA) was added into the flask during this time. After PbI_2_ was completely dissolved, the temperature was raised to 170 °C and was kept stirring in N_2_ gas for 20 min. At this state, 1.5 mL of as-prepared Cs-Oleate (preheated at 100 °C) was quickly injected into the PbI_2_ solution. About 5 s later, the reaction was quenched in an ice bath. After the flask was cooled down to room temperature, 20 mL of methyl acetate was added to precipitate the NCs. The NCs were then collected by centrifugation at 10,000 rpm for 10 min. After that, the supernatant was discarded, and the collected NCs were dispersed in 10 mL of Hexane. The solution then goes through another centrifuging step at 8000 rpm for 5 min. The precipitate is collected and kept in vacuum for 6 h to remove the residual solvents. The final NCs were dispersed in Hexane for further measurement and applications.

### Catalyst Preparation

The electrodes were prepared by mixing carbon black and catalysts with a mass ratio of 1 and painted on carbon fiber paper.

To specific, the electrode for the H-cell test was prepared through a drop-casting method. Firstly, 5 mg CsPbI_3_/rGO catalyst was dispersed in 1 mL hexane, and the catalyst ink was then mixed with 5 mg carbon black and ultrasonicated for 10 min. Finally, the obtained catalyst ink was drop-casted onto a carbon fiber paper (Toray Carbon Paper 090) and used as the electrode for the H-cell CO_2_ reduction test after drying in a vacuum desiccator for 5 h. For preparing the electrode for flow-cell test, 5 mg CsPbI_3_/rGO catalyst dispersed in 1 mL hexane, 5 mg Polytetrafluoroethylene (PTFE) powder (diameter of 1 μm) and 5 mg carbon black, then the obtained catalyst ink was sprayed onto a carbon fiber paper (AvCarb GDS2230) coated with PTFE and used as the electrode for flow-cell CO_2_ reduction test after drying in a vacuum desiccator for 5 h.

### Electrochemical CO_2_ Reduction Experiments

The electrochemical CO_2_ reduction experiments in H-cell were conducted in a three-electrode H-Cell reactor filled with CO_2_-saturated 0.1 M KHCO_3_ solution (Sigma Aldrich, 99.7%). The synthesized CsPbI_3_/rGO electrode was used as the working electrode, while Pt foil and Ag/AgCl electrodes were used as counter and reference electrodes, respectively. The working electrode and counter electrode were separated by a Nafion 117 membrane (Dupont) and connected to Autolab potentiostat (PGSTAT204). Each sample was evaluated for 1.5 h before the gas phase products were collected for analysis using gas chromatography (GC-2010, Shimadzu). The liquid phase products were analyzed by nuclear magnetic resonance (Avance III 600 MHz NMR, Bruker) using a pre-saturation method for water suppression.

The electrochemical CO_2_ reduction experiments in flow cell were conducted in a two-electrode flow-cell reactor with continuously pumped-in 1.0 M KHCO_3_ solution (Sigma Aldrich, 99.7%) at flow rate of 3 mL min^−1^ in the cathode (CsPbI_3_/rGO) side with 1.0 M KOH (Sigma Aldrich, 85.0%) in the anode (Ni foam) side. The CO_2_ gas continuously flowed through the back side of the cathode at flow rate of 50 mL min^−1^ via a custom-designed gas gate. The working electrode and counter electrode were separated by a Sustainion® anion exchange membranes and connected to Autolab potentiostat (PGSTAT204) equipped with a BOOSTER10A module. The gas phase and liquid phase products were collected for analysis using gas chromatography (GC-2010, Shimadzu) and nuclear magnetic resonance (Avance III 600 MHz NMR, Bruker) using the same method as the above-mentioned for the H-cell CO_2_ reduction test. The electrochemical impedance spectroscopy (EIS) was tested in CO_2_-saturated 0.1 M KHCO_3_ aqueous solution under 0 V vs RHE with frequency range of 0.01–10^5^ Hz.

### Characterization

Characterization was conducted to reveal the structure, optical and electrical properties of the synthesized materials. UV–visible absorbance spectrum of the samples was measured by a UV–visible spectrometer (Carry 60), and the photoluminescence spectrum was recorded with a Cary Eclipse Fluorescence Spectrophotometer. The TEM images were captured by a JEOL 2100 microscope operated at 200 kV. STEM-EDS was obtained using an EDS detector coupled with JEOL 2100 TEM. A Kratos AXIS Supra photoelectron spectrometer (He I radiation, hν = 21.22 eV) was used to measure the XPS spectra and the UPS energy state of the materials. The crystal structure of the NCs were measured by X-ray diffraction equipment (Rigaku Smartlab) using a monochromatic CuKα (λ = 0.154 nm) as a radiation source. Fourier transform infrared (FTIR) spectrum was recorded by a Bruker model Alpha-P FTIR with ATR accessory. Time-resolved photoluminescence (TRPL) was measured by an Edinburgh fluorescence spectrometer at room temperature. The photo-excited source was provided by a laser with exciting wavelength of 474 nm and a pulse of 82.4 ps. *In-situ* and *ex-situ* Raman spectroscopy was performed with a Renishaw inVia Qontor spectrometer with exciting wavelength of 514 nm.

### DFT Calculation

All the calculations for the optimization of CsPbI_3_ (001) plane and graphene-decorated CsPbI_3_ (001) were performed by using the density functional theory (DFT) method as implemented in the Vienna Ab initio Simulation Package (VASP). The Perdew–Burke–Ernzerhof (PBE) function of the generalized gradient approximation (GGA) was used for the calculation of electron exchange–correlation with the projector augmented wave (PAW) method [1–3]. Spin-polarization was also included through the calculations, and the cut-off energy of 500 eV for plain-wave basis sets was adopted. The convergence threshold was set to 10^–5^ eV, and 5 × 10^–3^ eV Å^−1^ for energy and force, respectively. The weak interaction was treated by DFT + D3 method using an empirical correction in Grimme’s scheme [4]. The reaction Gibbs free energy changes ($\Delta G$) for each elementary steps were calculated using the computational hydrogen electrode model shown in the following equation:

1 $$ \begin{array}{*{20}c} {\Delta G = \Delta {\text{E}} + \Delta {\text{ZPE}} - {\text{T}}\Delta {\text{S}} + \Delta G_{U} + \Delta G_{pH} } \\ \end{array} $$

where $\mathrm{\Delta E}$ is obtained directly from DFT calculations, $\mathrm{\Delta ZPE}$ is the change of zero-point energies (ZPE), T is the temperature of 298.15 K, and ΔS is the change in entropy of products and reactants. $\Delta {G}_{U}=-eU$, which is the contribution of the electrode potential to ΔG, and $\Delta {G}_{pH}=-{k}_{B}T\mathrm{ln}10\times pH$.

## Result and Discussion

### Growth of CsPbI_3_ on rGO

**Fig. 1:**
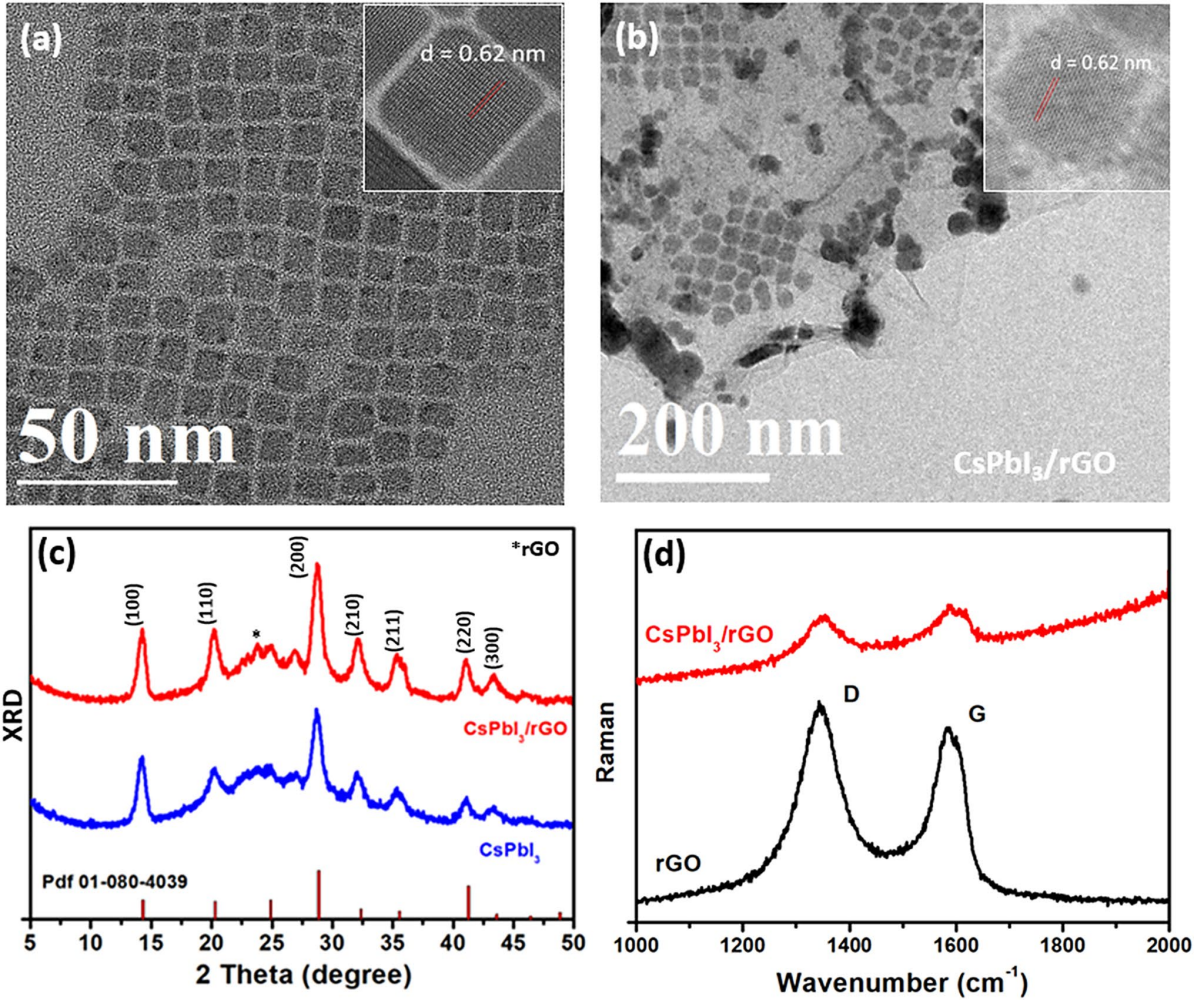
TEM images reveal the morphology of **a** CsPbI_3_ NCs and **b** CsPbI_3_/rGO composite (insets are the high-resolution TEM showing lattice fringe of the CsPbI_3_ NCs). **c** XRD pattens of CsPbI_3_ and CsPbI_3_/rGO composite together with the standard cubic phase CsPbI_3_ (PDF 01-080-4039). **d** Raman spectra of rGO and CsPbI_3_/rGO

CsPbI_3_ NCs were synthesized using a hot-injection synthetic method developed by Protesescu et al*.* [34]. To synthesize the CsPbI_3_/rGO composites, rGO was introduced into the PbI_2_ precursor solution before the injection of Cs-oleate precursor to initiate the growth of perovskite NCs. Previous studies have shown that perovskite materials have a strong binding affinity to rGO, leading to perovskite crystals readily growing and attaching onto the graphene surface [5, 29, 35]. Transmission electron microscopy (TEM) was used to reveal the structure of the NCs. Figure 1a shows the morphology of reference CsPbI_3_ NCs which are well dispersed cubic particles with an average size of $\sim$ 13 nm. The observed lattice spacing of $\sim$ 0.62 nm corresponds to (100) plane of cubic phase CsPbI_3_ perovskite as revealed in the high-resolution TEM image (inset of Fig. 1a). For the CsPbI_3_/rGO composite (Fig. 1b), the NCs are distributed on the rGO substrate with a slightly larger size and varying degrees of aggregations, implying that the presence of rGO can induce non uniform nucleation and growth of CsPbI_3_ NCs. Nevertheless, the CsPbI_3_ NCs grown on rGO still exhibit crystallinity of cubic phase perovskite. The existence and distribution of CsPbI_3_ perovskite on the rGO were also confirmed in the energy dispersive X-ray (EDX) mapping, which shows that Cs, Pb and I are well distributed on the rGO substrate (Fig. S1). In addition, the crystallinity of perovskite NCs was determined by X-ray diffraction (XRD). As seen in Fig. 1c, both CsPbI_3_ NCs and CsPbI_3_/rGO composite showed similar XRD patterns with characteristic peaks of cubic phase CsPbI_3_ perovskite (PDF No. 01-080-4039). The CsPbI_3_ NCs exhibited a preferential orientation at the (100) and (200) planes. In the CsPbI_3_/rGO composite, besides the peaks attributed to the cubic phase CsPbI_3_ perovskite, an additional peak at 23.2° was observed, which corresponds to the (002) plane of rGO [36].

Since the surface properties of the nanocrystals catalyst are critical for the electrocatalytic reaction [13, 37], we characterized the chemical composition and chemical state of the synthesized CsPbI_3_/rGO composite by X-ray photoelectron spectroscopy (XPS). The XPS survey scan clearly identified the C 1*s*, N 1*s*, O 1*s*, Cs 3*d*, Pb 4*f*, and I 3*d* peaks (Fig. S2). Fitting of the C 1*s* spectra has shown peaks at binding energies of 285.0, 286.2, 286.8 and 289.1 eV, which can be assigned to C–C, C=C, C–O and C=O bonds, respectively [38]. The C 1*s* features should originate from the surface ligands (oleic acid, oleylamine) used in the NCs synthesis and rGO. High resolution XPS of Cs, Pb and I are shown in Fig. S3. The fitted Cs 3*d* (725.4 and 739.3 eV), Pb 4*f* (139.1 and 143.8 eV) and I 3*d* (619.8 and 631.4 eV) peaks are consistent with the characteristic binding energy of CsPbI_3_ NCs [39]. Compared to the XPS spectra of CsPbI_3_, it shows that the introduction of rGO slightly alters the surface property of the CsPbI_3_ NCs (Fig. S3) with a small XPS peak shift (up to 0.4 eV) in all Cs 3*d*, Pb 4*f* and I 3*d* peaks, implying a change in the distribution of electrons on the surface of CsPbI_3_. The origin and consequence of this change will be discussed later. The existence of surface ligands of the NCs was also confirmed by FTIR measurement (Fig. S4). The FTIR spectra show the vibration peaks of the C–C and C–H stretching modes of the CH_2_ group and the C=O and O–H characteristic vibrations of the carboxylate ligand. There is a small increase in the intensity of C=O and O–H vibrations in the CsPbI_3_/rGO composite, which is possibly due to the contribution of rGO. All the above results have confirmed that the cubic phase CsPbI_3_ NCs have successfully grown on the rGO layers.

Raman spectroscopy is a powerful method to characterize carbon materials [40]. The pristine rGO shows two distinguishable peaks at 1350 (D band) and 1586 (G band) cm^−1^ (Fig. 1d) with the intensity ratio of *I*_D_/*I*_G_ ~ 1.16. The G band arises from the C–C stretching in the *sp*^2^-carbon networks and the D band originates from the presence of disorder or defects in the rGO [40, 41]. In the CsPbI_3_/rGO composite, the two characteristic peaks of D and G bands were also detected, validating the presence of rGO. Interestingly, the band ratio of *I*_D_/*I*_G_ is reduced to 1.08 in the CsPbI_3_/rGO composite. This implies that the loaded CsPbI_3_ NCs possibly passivate the defects on the surface of rGO.

### Optical Properties and Stability

**Fig. 2:**
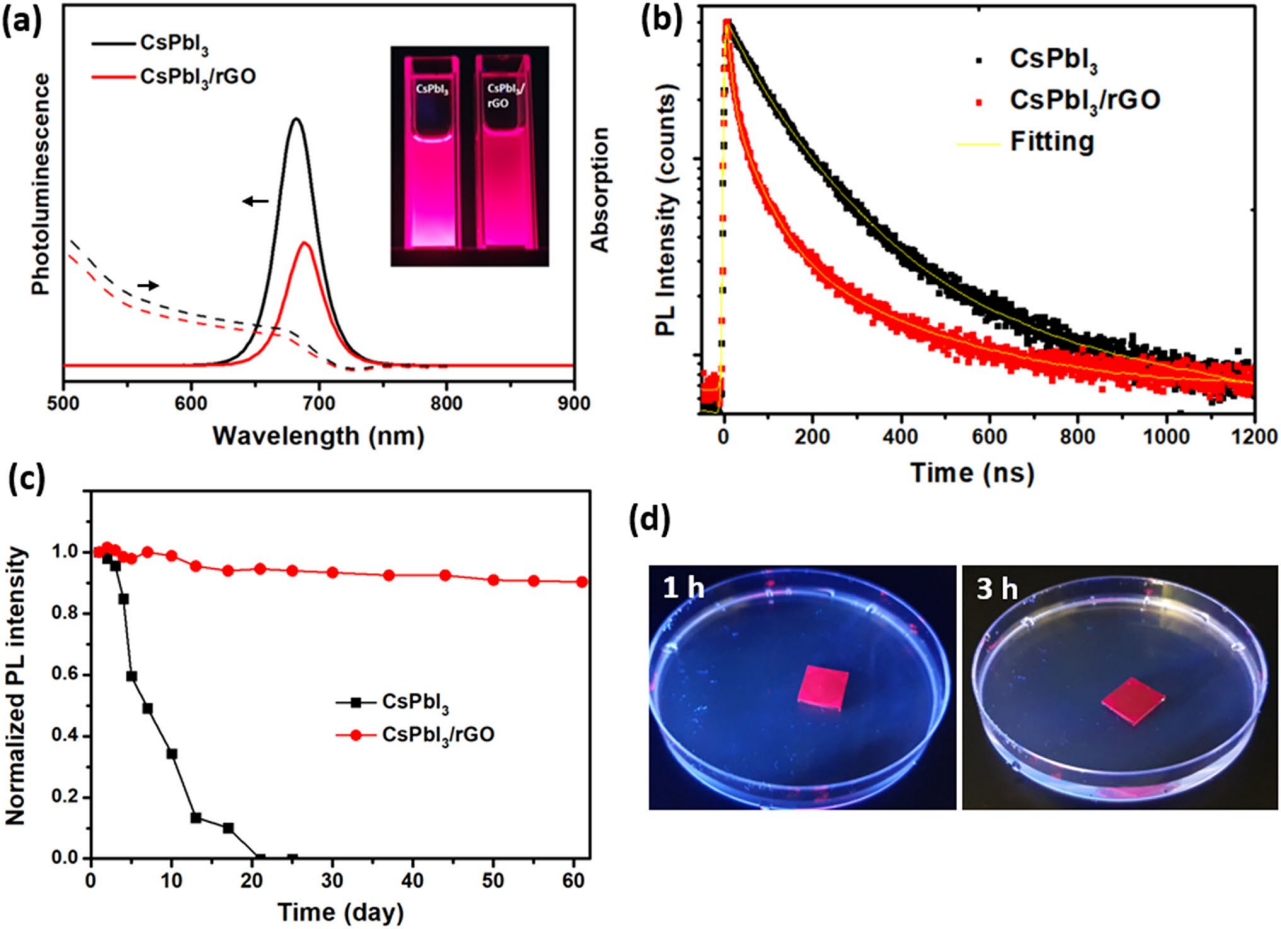
**a** PL and absorption spectra of CsPbI_3_ NCs and CsPBI_3_/rGO composite (insets are the photos of two solutions in hexane under UV excitation); **b** TR-PL decay spectra of CsPbI_3_ NCs and CsPBI_3_/rGO composite; **c** Plots of the change in PL intensity of CsPbI_3_ NCs and CsPBI_3_/rGO solutions during storage in ambient condition; **d** Photos showing the stability of CsPbI_3_/rGO thin film immersed in water for different durations. The film is exhibiting red emission under UV excitation. (Color figure online)

Perovskite nanocrystals are remarkable in terms of their light emitting properties which is closely related to the crystal structure and electronic properties of the materials [42]. Thus, optical properties of the as-synthesized perovskite NCs were measured to investigate the structural and electronic properties of the materials. As can be seen in Fig. 2a inset, the CsPbI_3_ NCs solution exhibits strong red emission under UV light (365 nm) excitation. The absorption and photoluminescence characteristics of the as-synthesized CsPbI_3_ (Fig. 2a) show an absorption onset of ~ 720 nm and an emission peak at 682 nm with full-width-at-half-maximum (FWHM) of 35 nm. The calculated band gap is ~ 1.71 eV (Fig. S5), which is typical for the cubic phase CsPbI_3_ perovskite [43]. By using a reference dye Rhodamine 6G [44, 45], we determined the photoluminescence quantum yield (PLQY) of the NCs to be ~ 73% (Table S1). In contrast, the CsPbI_3_/rGO exhibits much lower photoluminescence intensity (Fig. 2a) with a PLQY of 51%. A small red-shift in the absorption onset and PL photoemission peak position (688 nm) are also observed with the composite. The red-shift of 6 nm (~ 15.9 meV) could originate from the aggregation of NCs on the rGO sheets as observed in the TEM measurement. The lower PLQY implies that there is significant PL quenching in the material. This is confirmed by the time-resolved PL (TRPL) measurements (Fig. 2b). It shows that the presence of rGO has shortened the PL lifetime of the perovskite NCs. The pristine CsPbI_3_ NCs exhibit multi-exponential decays with an average lifetime of 62.1 ns, whereas the CsPbI_3_/rGO composite shows a 17% reduction of the average PL lifetime to 51.5 ns. Detailed calculation of the TRPL data is shown in Table S2. Apparently, addition of rGO has created an efficient charge transfer channel in the CsPbI_3_ NCs, which suppresses the radiative recombination of the excited excitons. Measurement of the energy band alignment of CsPbI_3_ and rGO by ultraviolet photoelectron spectroscopy (UPS) (Fig. S6a) has confirmed the feasibility for charge transfer between the two materials. The CsPbI_3_ NCs has a valence band energy edge at − 5.6 eV (vs vacuum) while rGO has a work function at − 5.2 eV, allowing an efficient hole transfer from CsPbI_3_ to rGO at the interface between the two materials (Fig. S6b).

Stability is a major problem for CsPbI_3_ NCs. The pristine NCs inks turned from dark brown to a light-yellow with yellow precipitate after 20 days storage in ambient condition (relative humidity 35% ~ 60%) (Fig. S7). This is resulted from the loss of surface ligands, formation of halide vacancy, and eventually the transformation of CsPbI_3_ NCs to PbI_2_ as reported previously [46, 47]. In contrast, the ink of CsPbI_3_/rGO still maintained dark brown colour even after 5 months of storage under the same condition (Fig. S7). The stability of both NCs was further assessed by the PL measurement as shown in Fig. 2c. A dramatical loss in PL intensity was observed in the pristine CsPbI_3_ NCs ink during storage, and the PL intensity dropped to zero in 20 days, whereas the CsPbI_3_/rGO composite ink still maintained more than 90% of its initial PL intensity after 2 months of storage. The XRD measurement shows that the CsPbI_3_/rGO composite largely retained its cubic phase after 2 months storage, with only a small portion of orthorhombic CsPbI_3_ detected (Fig. S8). We further tested the stability of the composite in water. Figure 2d shows the film of CsPbI/rGO composite still exhibited strong light emissions after being immersed in water for 3 h. The water contact angle measurement on the thin film of CsPbI_3_ and CsPbI_3_/rGO shows that the CsPbI_3_/rGO surface is more hydrophobic with a larger contact angle of 81° comparing to CsPbI_3_ film which shows a contact angle of approximate 73° (Fig. S9). Clearly, the rGO sheets provide significant protection to the CsPbI_3_ against aqueous environment.

### Catalytic CO_2_ Reduction Performance

**Fig. 3:**
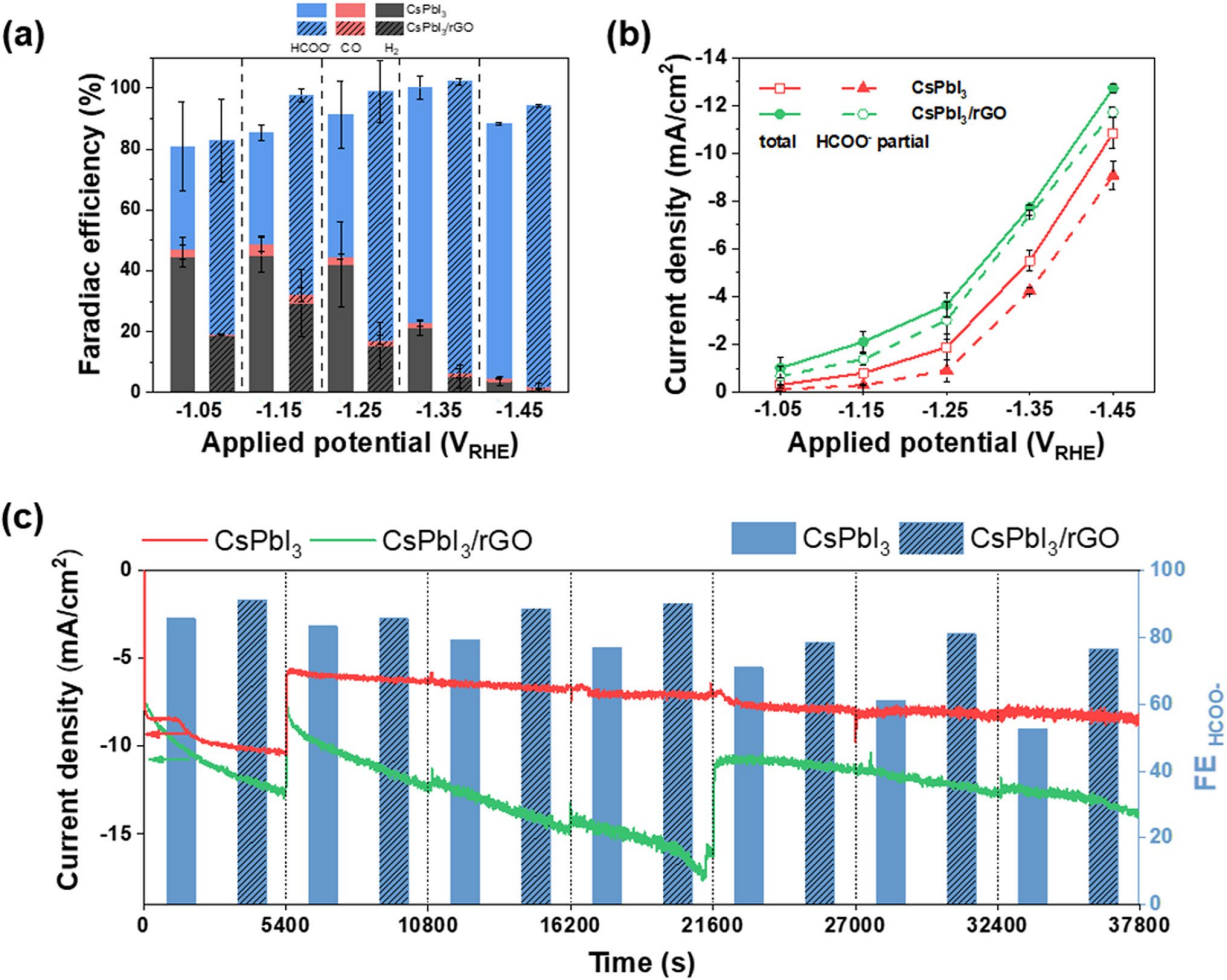
Electrochemical CO_2_ reduction performance of CsPbI_3_ and CsPbI_3_/rGO in CO_2_-saturated 0.1 M KHCO_3_. **a** FE of main products under different applied potentials. **b** Total CO_2_RR current density (solid symbols) and partial current density toward formate (dash symbols) under different applied potentials. **c** *I–t* curves and FE_formate_ in stability test under − 1.45 *V*_RHE_

To explore the CO_2_RR performance of the CsPbI_3_/rGO, the CO_2_RR experiments over CsPbI_3_ and CsPbI_3_/rGO were performed in a typical electrochemical H-cell configuration. The CO_2_RR experiments were conducted at different applied potentials ranging from − 1.05 to − 1.45 V vs reversible hydrogen electrode (RHE). The FE toward CO_2_ reduction products is shown in Fig. 3a. It shows that the CsPbI_3_/rGO catalysts has a higher FE_CO2RR_ under a more negative potential. The FE reaches to more than 90% at − 1.45 V vs RHE. Moreover, the CsPbI_3_/rGO catalysts exhibit a higher selectivity towards formate compared with that of the pristine CsPbI_3_ catalyst. It should be noted that formate is considered a promising hydrogen energy carrier because of its high volumetric hydrogen storage capacity, low toxicity and low flammability, thus efficient production of formate is desirable in practice [48]. A higher content of H_2_ was observed with the pristine CsPbI_3_ catalyst, which suggests that the interaction between the rGO and the CsPbI_3_ NCs suppresses the HER process. The gas phase products (i.e., CO and H_2_) were quantified by gas chromatography (GC) according to a calibration curve (Fig. S10). The excellent R^2^ value (R^2^ > 0.998) of the calibrate curve ensures the accuracy of the experimental results. The total current density and partial current density of formate are shown in Fig. 3b. The CsPbI_3_/rGO catalyst exhibits a total current density of 12.7 mA cm^−2^, in which the formate partial current density accounts for 11.7 mA cm^−2^. The electrochemical impedance spectroscopy (EIS) of the samples (Fig. S11) were measured to investigate the charge transfer resistance (*R*_ct_) of the catalysts in the presence of the CO_2_ saturated 0.1 M KHCO_3_ aqueous solution. The Nyquist plot of EIS was fitted according to the equivalent circuit shown in Inset of Fig. S11. Detail of the fitting results of the EIS spectrum is shown in Table S3. The much lower Rct of the CsPbI_3_/rGO composite indicates that the introduction of rGO into CsPbI_3_ has significant benefit for the electrochemical reaction of CO_2_RR.

The CO_2_RR performance of the CsPbI_3_/rGO catalyst was further evaluated in a 2-electrode flow-cell system. We achieved ~ 81.0 mA partial current toward CO_2_RR products over a 3 cm^2^ electrode. This is more than twofold of that in the H-cell system (Fig. S12). The CO_2_RR performance of the rGO was tested under the same condition (Fig. S13). However, no CO_2_RR product was detected with the bare rGO. Instead, H_2_ was the main product with FE ~ 100%. This implies that CsPbI_3_ is the main active material for the CO_2_ reduction in the composite.

We further tested the long-time stability of the catalysts at − 1.45 *V*_RHE_ for 10.5 h continuously. As shown in Fig. 3c, the CsPbI_3_ alone showed a significant deterioration on the selectivity to formate, with an FE_formate_ of only 52.5% at the end of test. In contrast, the CsPbI_3_/rGO maintained a FE_formate_ of 76.4% after 10.5 h continuous stability test. This confirms the improved CO_2_RR stability of the CsPbI_3_/rGO catalyst compared with bare CsPbI_3_. Moreover, the performance of our CsPbI_3_/rGO catalyst is competitive among other perovskite-based and metal-based catalysts for CO_2_ reduction reported in the recent literature such as sulfide-derived Pb or SnO_2_ porous nanowires catalyst (Table S4) [49, 50].

**Fig. 4:**
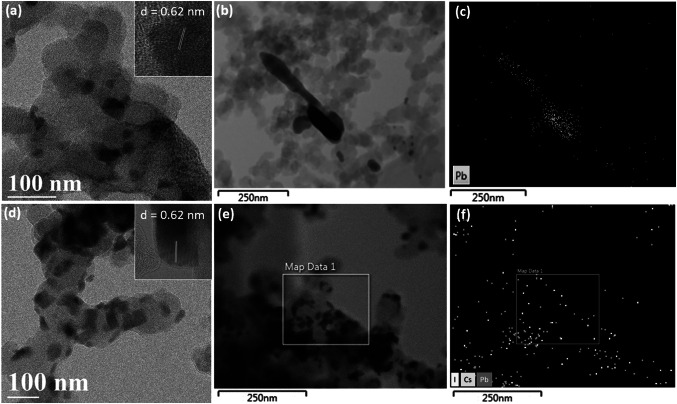
**a** TEM images of fresh electrodes CsPbI_3_; **b** TEM image of CsPbI_3_ after one EC CO_2_RR run under − 1.45 V_RHE_ and **c** the corresponding EDS elemental mapping image; **d** TEM image of fresh CsPbI_3_/rGO electrode; **e** TEM of CsPbI_3_/rGO electrode after one EC CO_2_RR run under − 1.45 *V*_RHE_ and **f** the corresponding EDS elemental mapping of composite for Cs (green), Pb (red), I (yellow) elements. (Color figure online)

To understand this phenomenon, we have analysed the morphology and composition of the fresh and the spent samples. As shown in Fig. 4a, the CsPbI_3_ without rGO appear in the form of nanoparticles with well-defined crystalline structure dispersed in a carbon matrix. After the first cycle of EC CO_2_RR reaction under − 1.45 *V*_RHE_, the NCs of CsPbI_3_ without rGO transformed to Pb aggregations (Fig. 4b), as evidenced by the TEM EDX mapping (Fig. 4c). In case of CsPbI_3_ with rGO, the CsPbI_3_/rGO catalyst also exhibit the morphology of well-crystalline particles dispersed in a carbon matrix (Fig. 4d), but this morphology remained intact after the same EC CO_2_RR condition (Fig. 4e). From the EDS measurement (Fig. 4f), it can be seen that the chemical composition of CsPbI_3_ NCs in the CsPbI_3_/rGO was well maintained. The obtained atomic percentages of C, Cs, Pb and I were 98.4%, 0.34%, 0.34% and 0.92%, respectively, corresponding to a Cs/Pb/I ratio of approximately 1/1/2.7, which is close to that of pristine CsPbI_3_ perovskite phase. After the long-time stability test, despite of the emergence of nanoparticles, the CsPbI_3_/rGO still preserved the original crystal phase and chemical compounds (Fig. S14), as opposed to the pristine CsPbI_3_ catalyst which completely changed to Pb particles (Fig. S15). It is noted that the aggregation of CsPbI_3_ NCs in the CsPbI_3_/rGO composite led to the formation of nanorods after the long reaction time, which is expected to reduce the surface area of the catalyst and adversely affect the overall electrocatalytic performance.

**Fig. 5:**
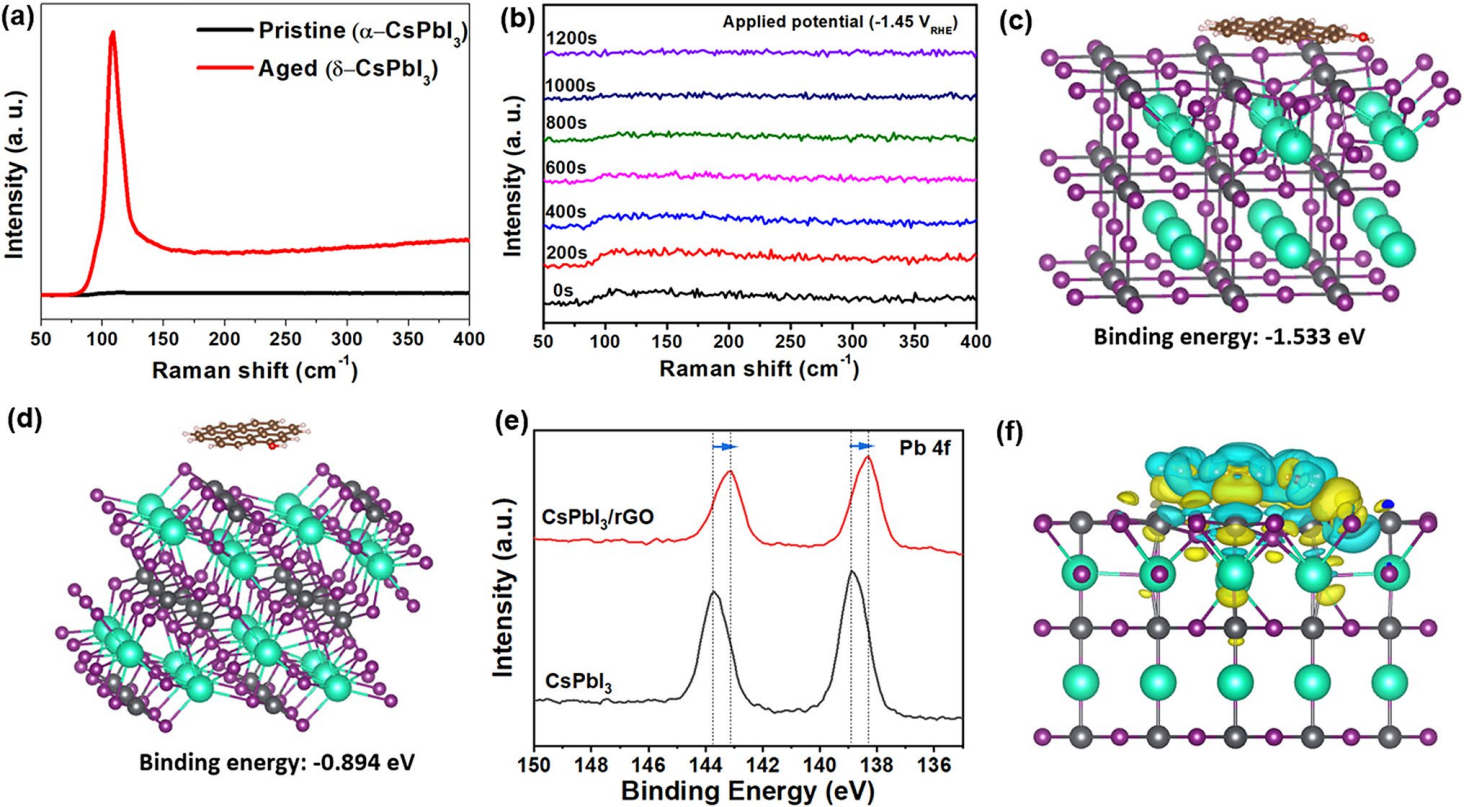
**a** Raman spectra of pristine CsPbI_3_ (black) and aged CsPbI_3_ (red); **b** *In-situ* Raman spectra of CsPbI_3_/rGO under EC CO_2_RR conditions with applied potential of − 1.45 *V*_RHE_; The DFT models of $\alpha$-CsPbI_3_ **c** and $\delta$-CsPbI_3_ **d** with rGO supported and the corresponding calculated binding energy. **e** XPS Pb 4*f* spectra of fresh CsPbI_3_ and CsPbI_3_/rGO electrodes. **g** Electron transfer between $\alpha$-CsPbI_3_ and rGO from DFT calculations, where yellow area means charge accumulation, and cyan area means charge depletion. (Color figure online)

The structural stability of the samples was further investigated by *in-situ* Raman spectroscopy, which is a powerful method to monitor phase transformation of materials. The pristine CsPbI_3_ ($\boldsymbol{\alpha }$-CsPbI_3_) exhibited an indiscernible Raman peak (Fig. 5a), which is consistent with the previous study that $\boldsymbol{\alpha }$-CsPbI_3_ only shows Raman features at a low frequency region [51]. After being aged in the ambient condition, an obvious vibration mode appeared at 108 cm^−1^, which matches the characteristic fingerprint of ${\varvec{\delta}}$-CsPbI_3_, implying that the perovskite undergoes a phase transformation from $\boldsymbol{\alpha }$-CsPbI_3_ phase to ${\varvec{\delta}}$-CsPbI_3_ phase [51]. The in-situ Raman spectroscopy was used to track phase transition during the EC reaction of CsPbI_3_/rGO catalyst. As shown in Fig. 5b, there is no change in the Raman spectra of the CsPbI_3_/rGO catalyst under − 1.45 V_RHE_ EC CO_2_RR conditions in the 1200 s testing period, indicating that the phase transformation to ${\varvec{\delta}}$-CsPbI_3_ did not occur and the CsPbI_3_ in CsPbI_3_/rGO remained in the α phase.

We carried out density functional theory (DFT) calculations of binding energy of rGO with $\boldsymbol{\alpha }$**-** and ${\varvec{\delta}}$**-**CsPbI_3_ to understand the improved stability of CsPbI_3_/rGO. As shown in Fig. 5c, d, the binding energy of rGO with $\boldsymbol{\alpha }$-CsPbI_3_ (black phase) is − 1.533 eV, which is significantly lower than that of rGO with *δ*-CsPbI_3_ (yellow phase) (− 0.894 eV), suggesting that the CsPbI_3_ NCs formed on rGO sheets preferably maintains the $\boldsymbol{\alpha }$-CsPbI_3_ phase thermodynamically. This also explains the enhanced stability of the $\boldsymbol{\alpha }$-CsPbI_3_ in the CsPbI_3_/rGO composite compared to the pristine CsPbI_3_ NCs. In addition, comparing the XPS spectra of fresh CsPbI_3_ and CsPbI_3_/rGO electrodes (Figs. 5e and S16), we have observed a distinct shift toward lower-binding energy in the XPS spectra of Pb 4*f*, Cs 3*d* and I 3*d* of CsPbI_3_/rGO. This indicates an increased electron density in the CsPbI_3_ material in the presence of rGO. The observation agrees well with the DFT calculation results (Fig. 5f), which show electron transfer between $\boldsymbol{\alpha }$-CsPbI_3_ and rGO, and the electron tends to accumulate around the $\boldsymbol{\alpha }$-CsPbI_3_. The unique charge distribution in CsPbI_3_/rGO material plays an important role in the EC CO_2_RR performance of the material as discussed below.

### Reaction Pathway

**Fig. 6:**
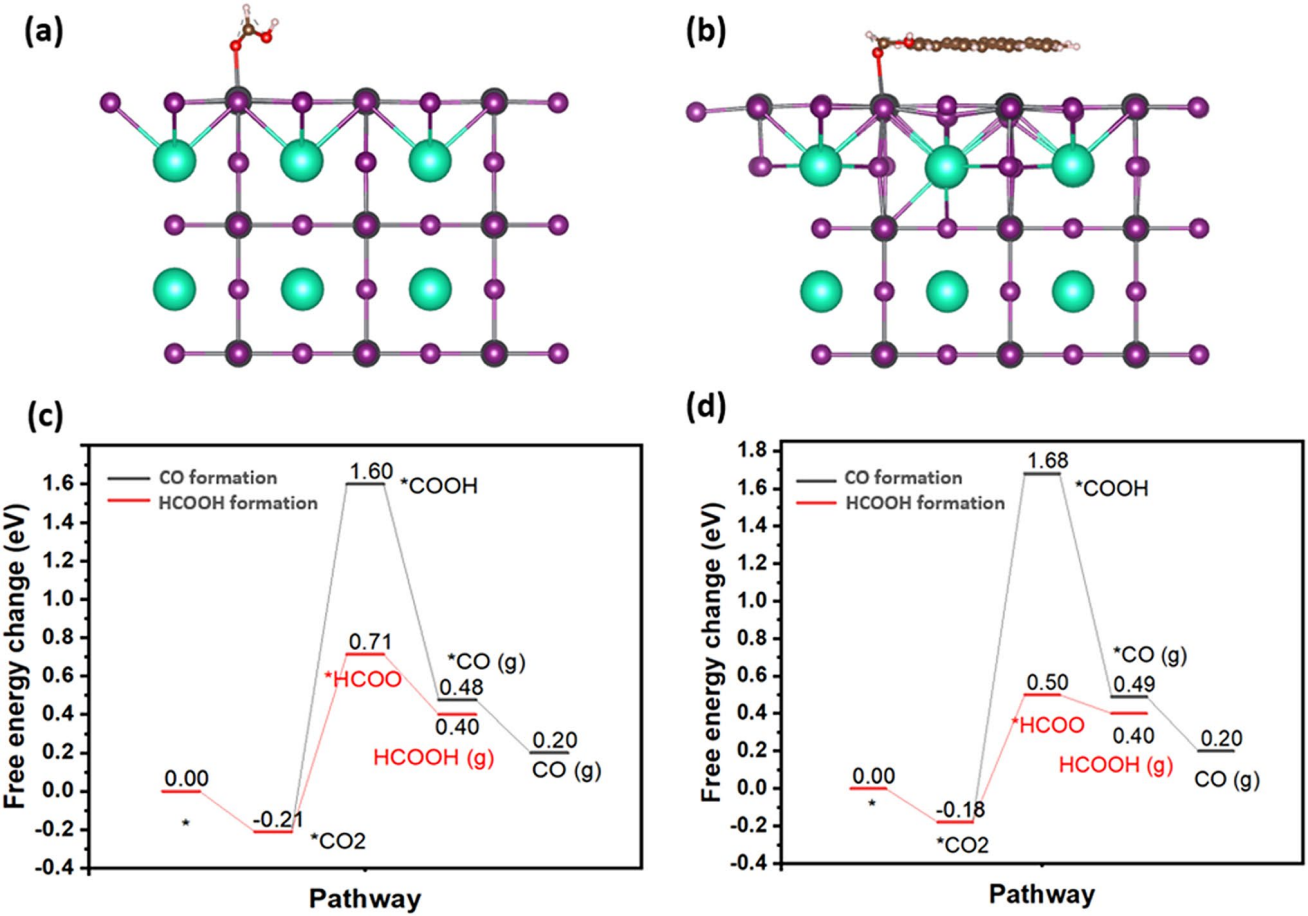
Models of **a** CsPbI_3_ and **b** CsPbI_3_/rGO composite with HCOOH species in the DFT calculations; **c, d** Free-energy diagrams showing the CO and HCOOH formation pathways in the CO_2_ reduction reaction when **c** CsPbI_3_ and **d** CsPbI_3_/rGO catalyst were used

The above results show that the integration of rGO to CsPbI_3_ significantly improves the performance, selectivity, and stability of $\boldsymbol{\alpha }$-CsPbI_3_ phase in EC CO_2_ reduction. To understand the catalysis mechanism, we calculated the activation energy of adsorbed species formed in the CO_2_ reduction reaction on CsPbI_3_ (100) facet with and without rGO, since this is the dominant phase in our material as shown in the XRD (Fig. 1c). Previous theoretical investigations have suggested that Cs cannot be the active site because Cs–C bonding is less favoured than Cs–O bonding [20]. In contrast, the Pb–C bonding is favourable to form. Therefore, Pb is selected as the active site in the DFT calculations. The activation energy of adsorbed species on Pb site was calculated on models of $\boldsymbol{\alpha }$-CsPbI_3_ with and without rGO supported as illustrated in Fig. 6a, b. The resulting free-energy diagrams are shown in Fig. 6c, d, which include two possible pathways for CO_2_ reduction, i.e., (i) reduction of CO_2_ to the COOH intermediate toward CO and (ii) reduction of CO_2_ to the *HCOO intermediate toward HCOOH. The formation of *HCOO intermediate is thermodynamically favourable according to the simulations. This is consistent with the experimental result of high selectivity toward HCOO^−^ product rather than CO product. Interestingly, when CsPbI_3_ is anchored on rGO, the activation energy barrier for *HCOO intermediate formation is reduced from 0.92 to 0.68 eV. It means the CsPbI_3_/rGO composite can further facilitate the reaction toward the formate production. This is attributed to the electron accumulation formed around Pb atoms in the presence of rGO (Fig. 5f), which strengthens the Pb–C bonding and the adsorption of *H species, followed by the acceleration of the protonation of CO_2_ for formate formation. It should be noted that the HER is not preferred in both material systems due to the high energy barrier according to our DFT simulation (Fig. S17).

Additionally, we have observed the formation of Pb metal in the spent CsPbI_3_ catalyst (Fig. 4b). It suggests that the CsPbI_3_ particles slowly lost Pb atoms during the CO_2_ reduction reaction. Therefore, a model of CsPbI_3_ with Pb vacancy was also built to simulate the CO_2_ reduction reactions. The calculation shows that with Pb vacancy in the CsPbI_3_ crystal, the energy barrier for *HCOO intermediate formation increases to 1.31 eV (Fig. S18). This explains the decrease of formate selectivity during the long-term stability test in CsPbI_3_ catalyst. Our results have demonstrated an effective strategy to prevent the phase degradation or Pb defect formation in CsPbI_3_, which is critical for maintaining the superior CO_2_RR performance and formate selectivity.

## Conclusion

In summary, we have devised an efficient CsPbI_3_/rGO composite catalyst for CO_2_RR in aqueous electrolyte. Compared to the pristine CsPbI_3_ NCs, the α-CsPbI_3_ perovskite structural degradation in CsPbI_3_/rGO composite is thermodynamically mitigated, hindering the formation of less-active Pb defects or even Pb particles. Furthermore, by redistributing electrons near the CsPbI_3_/rGO interface, the rGO lowers the energy barrier for the formation of *HCOO intermediates and protonation process in the CsPbI_3_/rGO catalyst, thus promoting the CO_2_RR pathway toward formate product with a high selectivity (> 90% FE_formate_) at applied voltage of − 1.45 V vs RHE. The study has revealed the perspective application of metal halide perovskite material in electrochemical CO_2_RR while paves the way for the development of selective, stable, and active new catalysts.

### Supplementary Information

Below is the link to the electronic supplementary material.

Supplementary file1 (PDF 1461 KB)
