# Supplementary material for: Efficient CO2 Reduction to Formate on CsPbI3 Nanocrystals Wrapped with Reduced Graphene Oxide
Source: Nanomicro Lett. 2023 Jun 29;15:161. doi: 10.1007/s40820-023-01132-3 (PMC10310658; doi:10.1007/s40820-023-01132-3)
Supplement: Supplementary file 1 — Supplementary file1 (PDF 1461 KB) [file 40820_2023_1132_MOESM1_ESM.pdf]

Supporting Information for

Efficient CO_2 Reduction to Formate on $CsPbI_3$ Nanocrystals Wrapped with Reduced Graphene Oxide

Minh Tam Hoang[1, 2, ‡], Chen Han[3, ‡], Zhipeng Ma[3, ‡], Xin Mao[1, 2, ‡], Yang Yang[1, 2], Sepideh Sadat Madani[1, 2], Paul Shaw[4], Yongchao Yang[3], Lingyi Peng[3], Cui Ying Toe[3, 5], Jian Pan[3], Rose Amal[3], Aijun Du[1, 2], Tuquabo Tesfamichael[1, 2], Zhaojun Han[3, *], Hongxia Wang[1, 2, *]

[1] School of Chemistry and Physics, Faculty of Science, Queensland University of Technology, Brisbane, QLD 4001, Australia

[2] Centre for Materials Science, Queensland University of Technology, Brisbane, QLD 4001, Australia

[3] School of Chemical Engineering, The University of New South Wales, Kensington, NSW 2052, Australia

[4] Centre for Organic Photonics & Electronics (COPE), School of Chemistry and Molecular Biosciences, The University of Queensland, Brisbane 4072, Australia

[5] School of Engineering, The University of Newcastle, Callaghan, NSW 2038, Australia

[‡] Minh Tam Hoang, Chen Han, Zhipeng Ma, and Xin Mao equally contributed to this work.

*Corresponding authors. E-mail: hx.wang@qut.edu.au (H.W.), zhaojun.han@unsw.edu.au (Z. H.)

Supplementary Figures and Tables

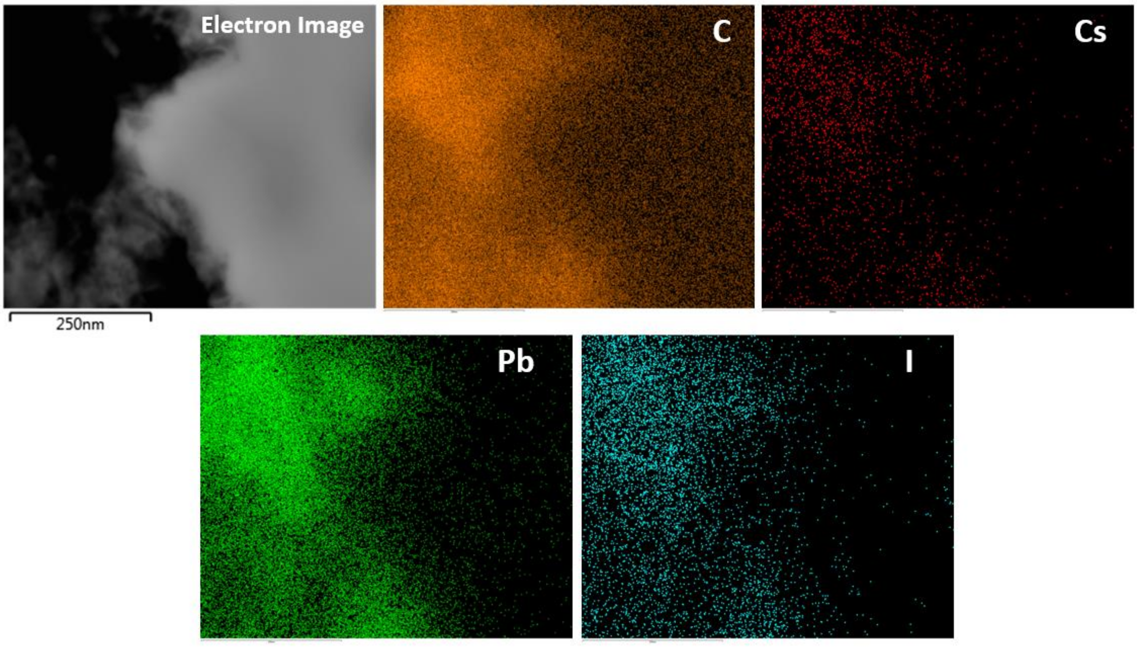

Fig. S1 EDX elemental mapping of $CsPbI_3$/rGO composite showing the distribution of Cs, Pb and I on the C matrix

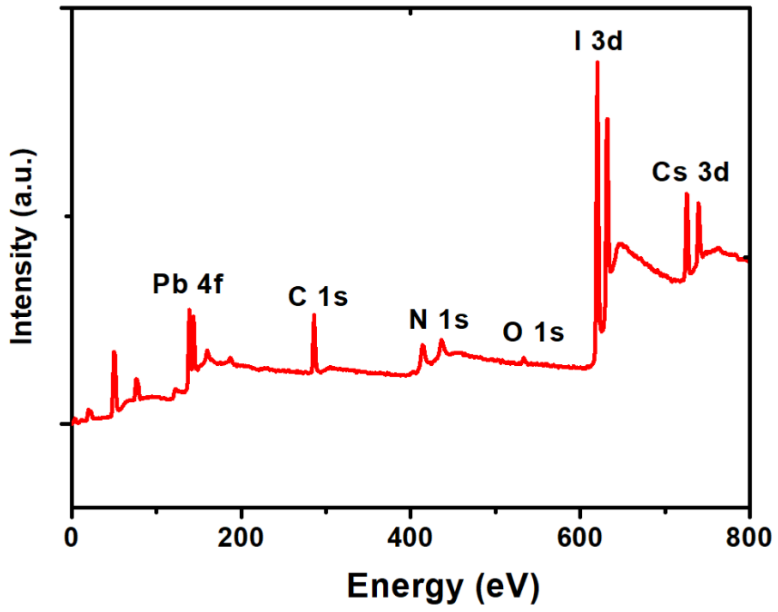

Fig. S2 XPS survey scan of $CsPbI_3$/rGO composite

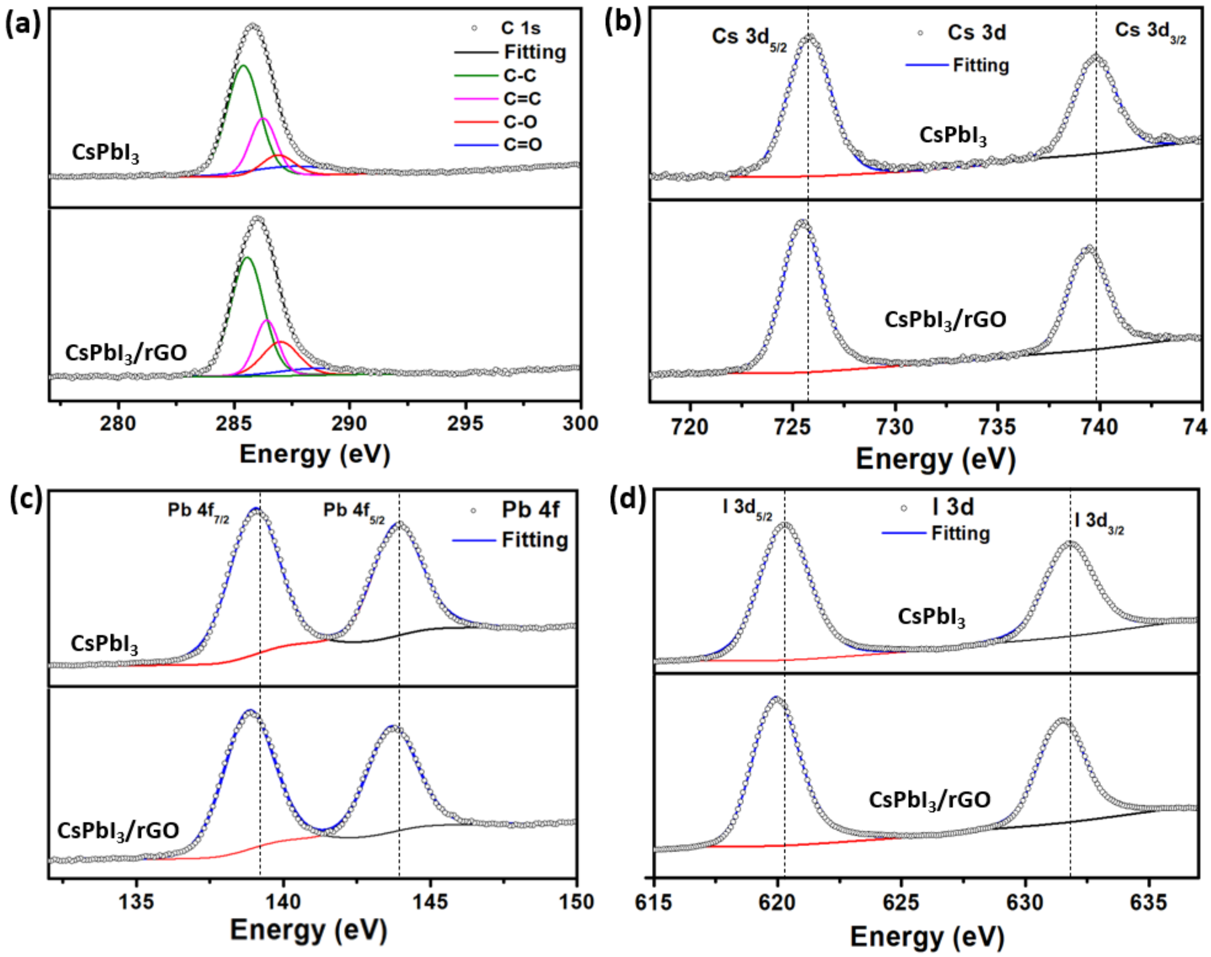

Fig. S3 XPS spectra of $CsPbI_3$ and $CsPbI_3$/rGO NCs showing characteristic peaks of Cs 3d, Pb 4f, I 3d and C 1s

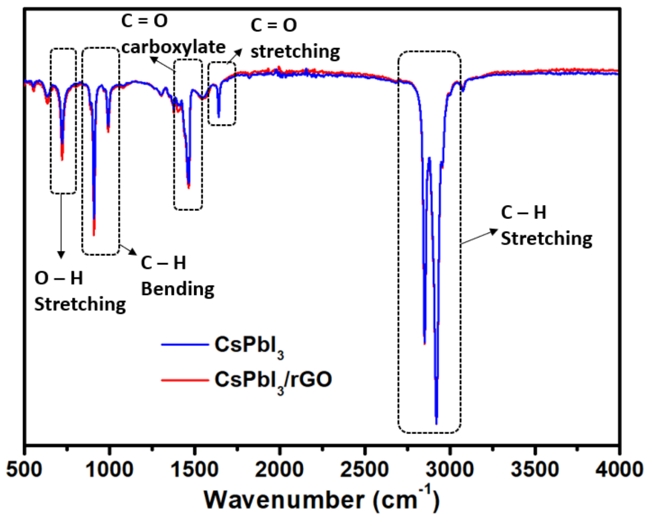

Fig. S4 Fourier-transform infrared spectra of $CsPbI_3$ and $CsPbI_3$/rGO showing clear vibration peaks of surface ligand

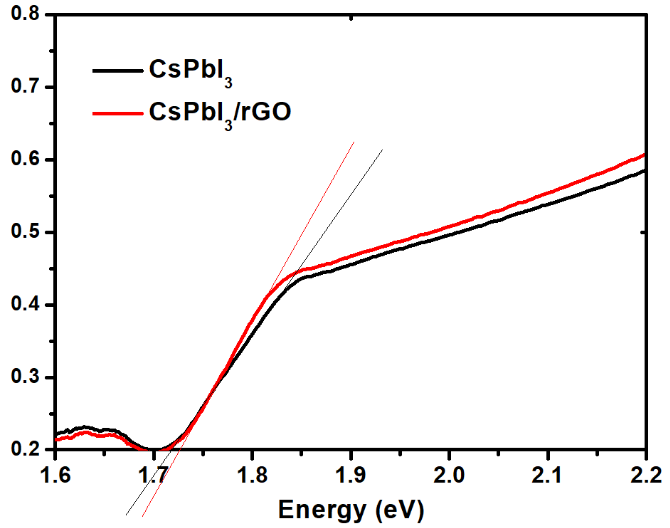

Fig. S5 Tauc plot showing the bandgap of $CsPbI_3$ NCs and $CsPbI_3$/rGO composite

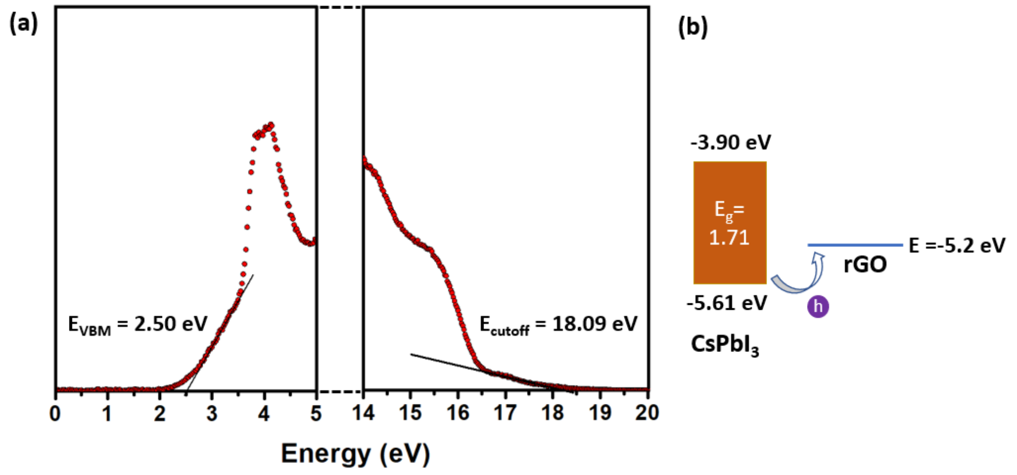

Fig. S6 (**a**) UPS measurement of $CsPbI_3$ NCs showing fitting for valance band maximum and cut-off energy; (**b**) Illustration of energy band alignment between $CsPbI_3$ and rGO calculated from UPS and band gap measurement. With $E_{valance\ band} = E_{HeI} + E_{cutoff} - E_{VBM}$

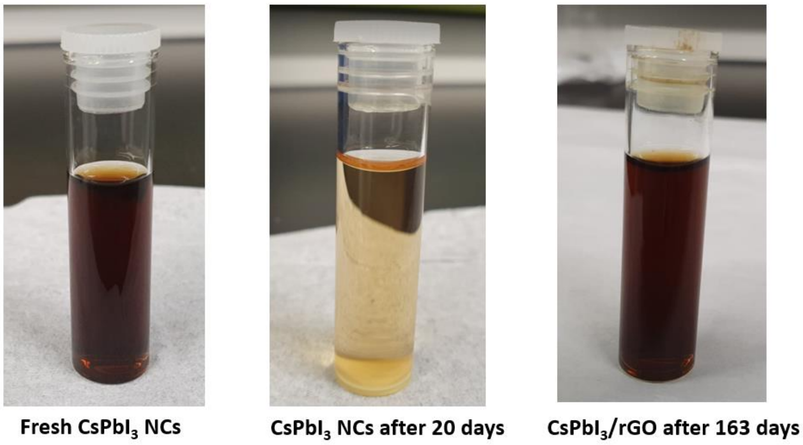

Fig. S7 Picture showing solution of $CsPbI_3$ and $CsPbI_3$/rGO in hexane after storage in ambient condition for long time

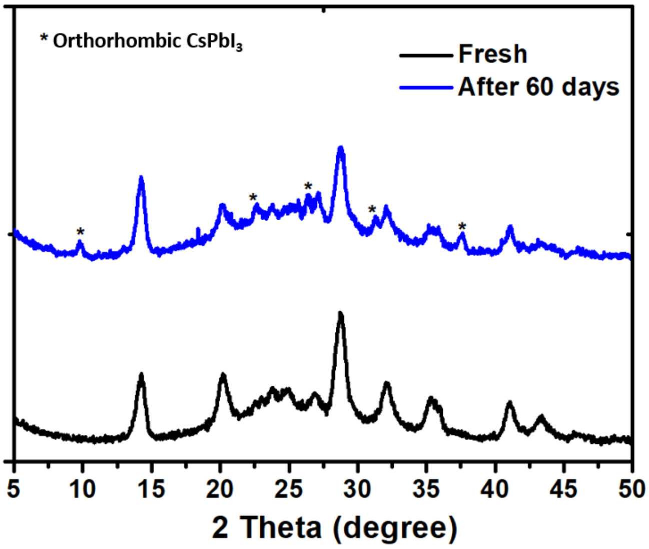

Fig. S8 XRD spectra of $CsPbI_3$/rGO composite after keep in ambient condition for 60 days, in comparison with the XRD of the fresh samples

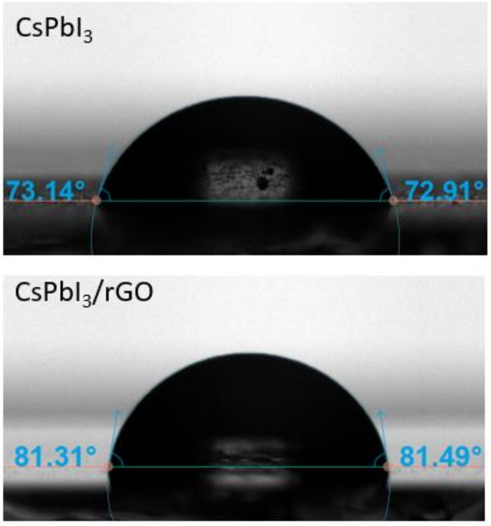

Fig. S9 Water contact angle measurement of $CsPbI_3$ NCs film and $CsPbI_3$/rGO film on glass substrate

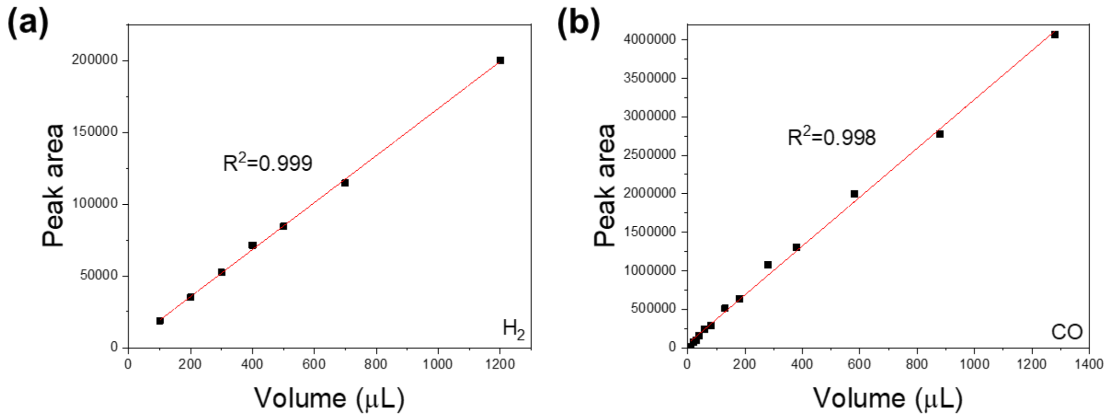

Fig. S10 GC calibration curves of (**a**) H_2 and (**b**) CO

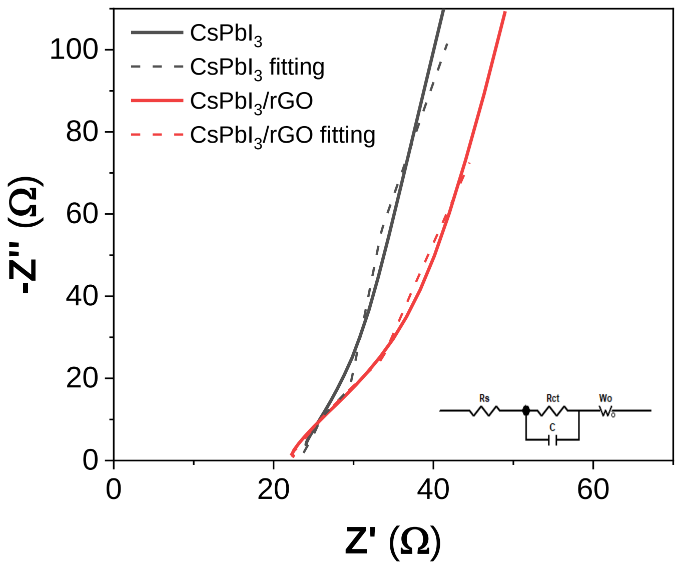

Fig. S11 EIS spectra of $CsPbI_3$ and $CsPbI_3$/rGO in CO_2 saturated 0.1 M $KHCO_3$ under 0 V_{RHE} and fitting graph of EIS spectra

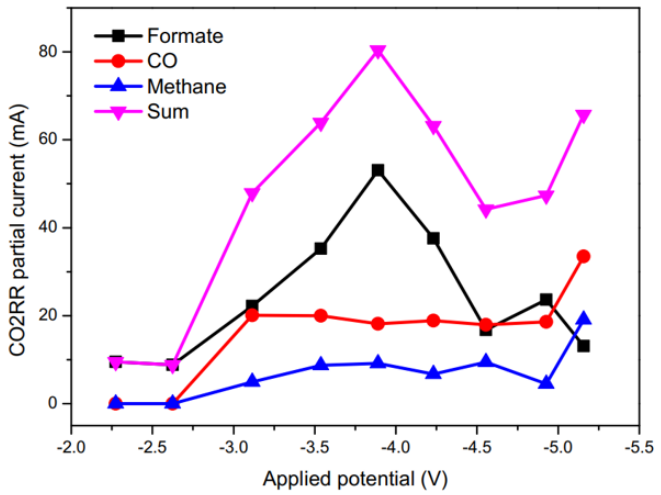

Fig. S12 The CO_2RR performance of the $CsPbI_3$/rGO catalyst in a 2-electrode flow-cell system

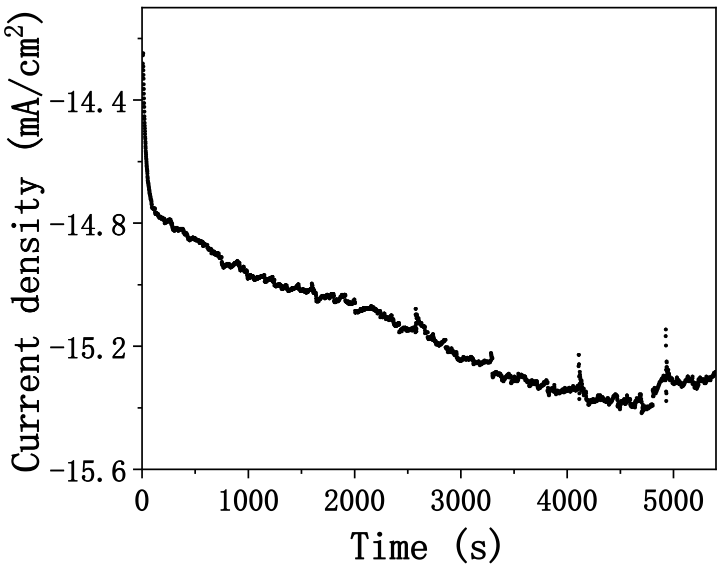

Fig. S13 The performance of rGO catalyst under CO_2RR conditions at -1.45 V $_{RHE}$

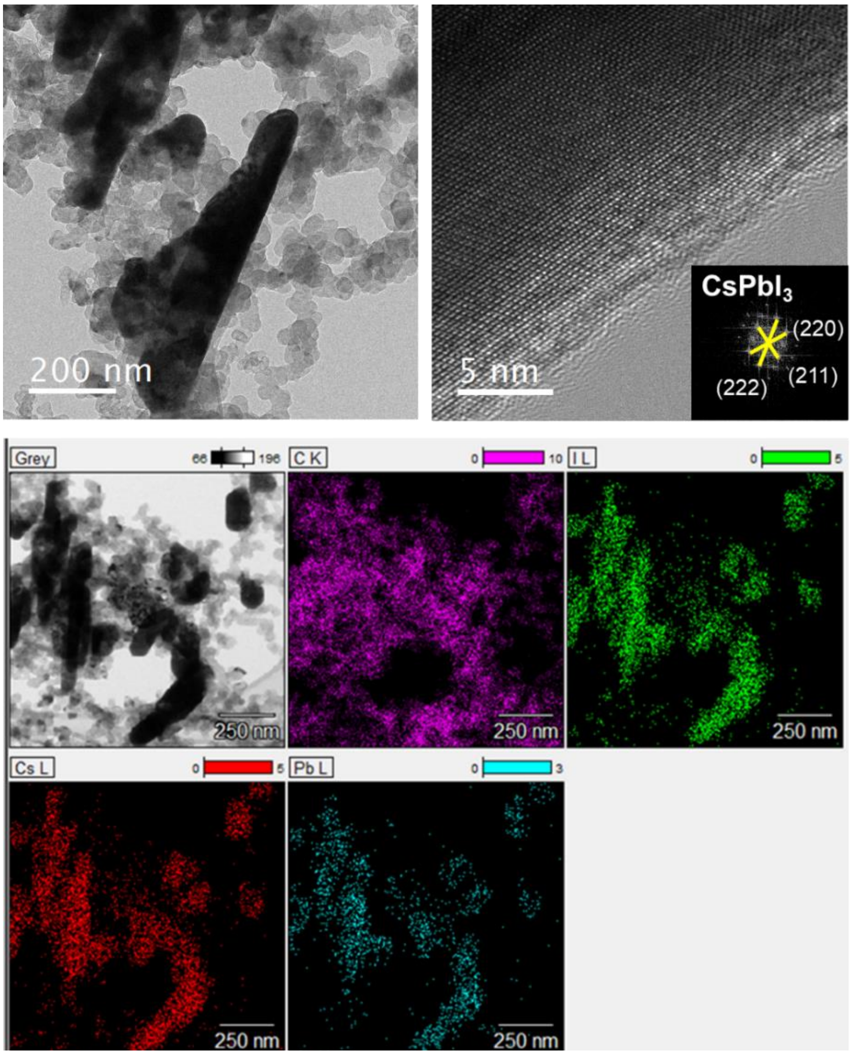

Fig. S14 TEM images and corresponding EDX elemental mapping of $CsPbI_3$/rGO after stability test under -1.45 V_{RHE}

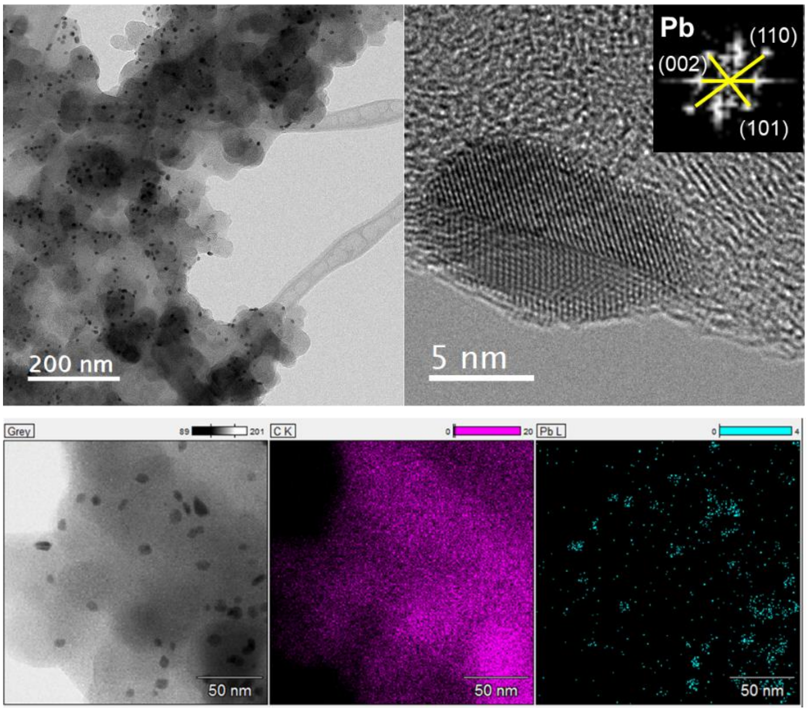

Fig. S15 TEM images and corresponding EDX elemental mapping of $CsPbI_3$ after stability test under -1.45 V_{RHE}

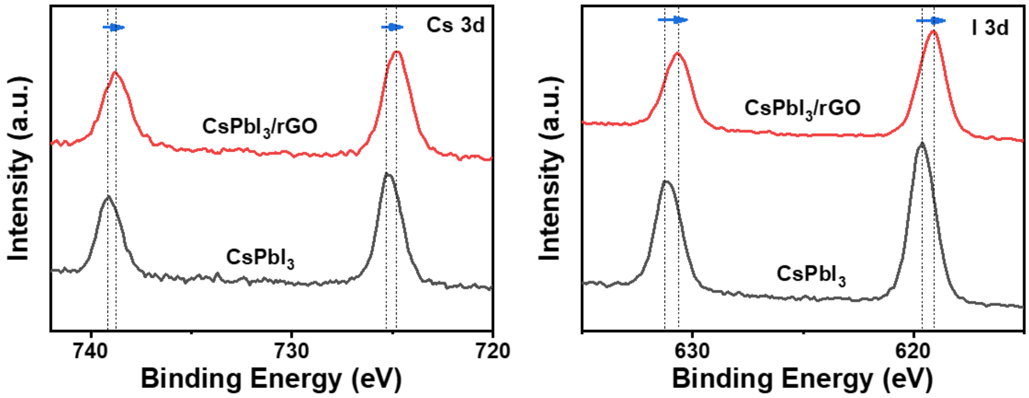

Fig. S16 XPS Cs 3d and I 3d spectra of fresh electrodes with $CsPbI_3$ and $CsPbI_3$/rGO

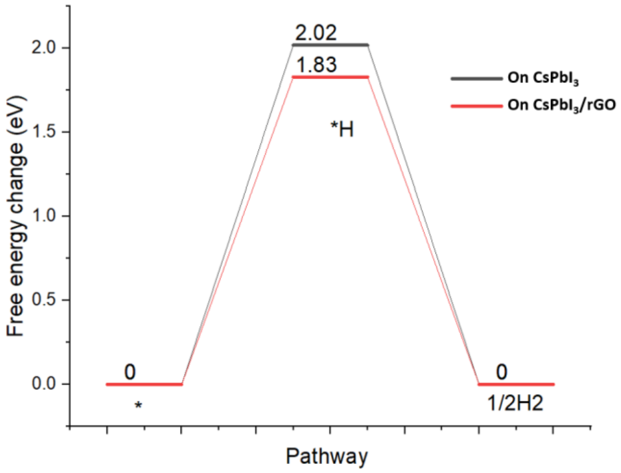

Fig. S17 Free-Energy Diagrams for the energetics of HER process when $CsPbI_3$ and $CsPbI_3$/rGO were used

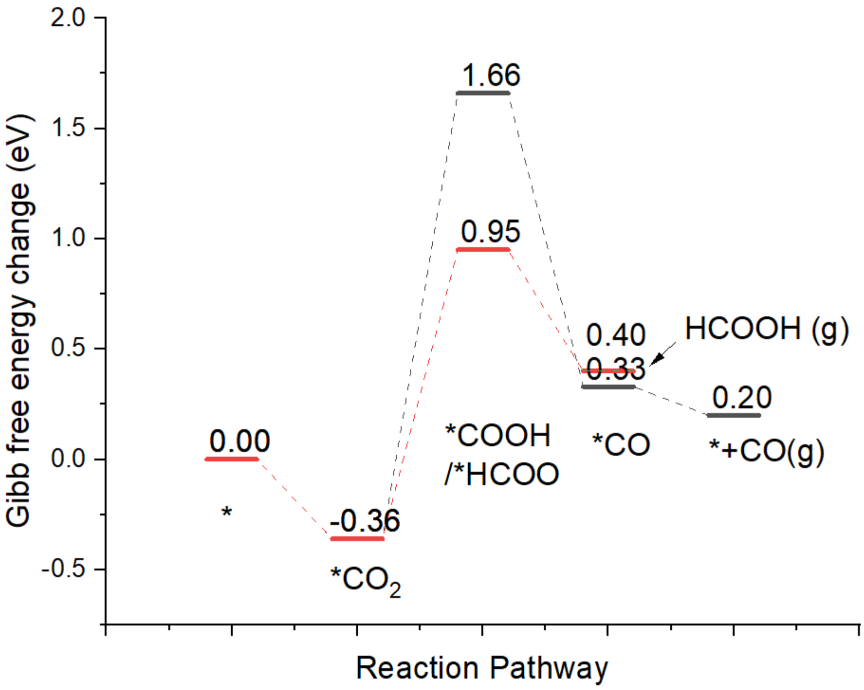

Fig. S18 Free-Energy Diagrams for the energetics of CO_2 reduction process when $CsPbI_3$ with Pb vacancy defects was used

Table S1 Relative PLQY detail calculation using Rhodamine 6G as reference dye [S1, S2]

$Q_x = Q_R \frac{I_x}{I_R} \frac{A_R}{A_x} \frac{n_x^2}{n_R^2}$	**Absorbance (at 350 nm)**	**Integrated PL intensity**	**FWHM (nm)**	**PLQY (%)**
**Rhodamine 6G**	0.098	27787.82	34.49	95.0
**$CsPbI_3$**	0.101	21572.05	34.96	73.0
**$CsPbI_3$/rGO**	0.094	13878.30	38.70	50.5

Table S2 Fitted TR-PL data of $CsPbI_3$ and $CsPbI_3$/rGO

**Sample**	**τ_1 (ns)**	**A_1 (%)**	**τ_2 (ns)**	**A_2 (%)**	**τ_3 (ns)**	**A_3 (%)**	**τ_{ave} (ns)**
**$CsPbI_3$**	11.508	15.0	28.659	61.9	93.603	23.1	62.11
**$CsPbI_3$/rGO**	12.546	40.6	61.100	37.5	2.050	21.9	51.48

The PL decay was fitted with a tri-exponential decay function [S3]:

$$I(t) = \sum_{i=1}^{3} A_i \exp\left(-\frac{t}{\tau_i}\right)$$

Table S3 Fitted data of the EIS measurement in Fig. S11

	**Rs (Ω)**	**Rct (Ω)**	**Wo-R (Ω)**
**$CsPbI_3$**	23.06	111.3	23.91
**$CsPbI_3$/rGo**	21.37	60.91	38.1

Table S3 Comparison of the performance of our developed $CsPbI_3$/rGO catalyst with other recently reported other perovskite-based and metal-based catalyst for electrochemical reduction of CO_2

	**Sample**	**Products**	**Max. FE, %**	**Stability**	**Current Density (mA/cm^2)**	**Refs.**
**Perovskite**	$CsPbI_3$/rGO	$HCOO^-$	95.9	10.5 h, FE_{HCOO-} 76.4%	12.7	This work
	$CsPbBr_3$ nanocrystals	CH_4, CO	32 for CH_4, 40 for CO	350 h		[S4]
	Cs_2PdBr_6	CO	78	10 h		[S5]
	$Cs_3Bi_2Br_9$	HCOOH	80	20 h		[S6]
	$La_{0.5}Ba_{0.5}CoO_3$	$HCOO^-$	99	60 h, FE_{HCOO-} 90%		[S7]
	La_2CuO_4	C_2H_4	40.3	-		[S8]
**Pb-based catalysts**	Sulfide-derived (SD)-Pb	$HCOO^-$	88	-	12	[S9]
	Pb-MOF	HCOOH	96.8	-		[S10]
	Sn–Pb	$HCOO^-$	79.8	-		[S11]
	Pd_3Bi	$HCOO^-$	~ 100	-		[S12]
**Sn, Bi and In-based catalysts**	Sn-pNWs	HCOOH	80	-		[S13]
	Bi nanodendrites	$HCOO^-$	96.4	10 h	15.2	[S14]
	Sulfur-doped indium	$HCOO^-$	93	-		[S15]

Supplementary References

[S1] D. Magde, R. Wong, P.G. Seybold, Fluorescence quantum yields and their relation to lifetimes of rhodamine 6G and fluorescein in nine solvents: Improved absolute standards for quantum yields. Photochem. Photobiol. **75**, 327-334 (2002). https://doi.org/10.1562/0031-8655(2002)075<0327:FQYATR>2.0.CO;2

[S2] G.A. Crosby, J.N. Demas, Measurement of photoluminescence quantum yields. Review. J. Phys. Chem. C **75**, 991-1024 (1971). https://doi.org/10.1021/j100678a001

[S3] W. Chen, N.D. Pham, H. Wang, B. Jia, X. Wen, Spectroscopic Insight into efficient and stable hole transfer at the perovskite/spiro-OMeTAD interface with alternative additives. ACS Appl. Mater. Interfaces **13**, 5752-5761 (2021). https://doi.org/10.1021/acsami.0c19111

[S4] K. Chen, K. Qi, T. Zhou, T. Yang, Y. Zhang et al., Water-dispersible $CsPbBr_3$ perovskite nanocrystals with ultra-stability and its application in electrochemical CO_2 reduction. Nano-Micro Lett. **13**, 172 (2021). https://doi.org/10.1007/s40820-021-00690-8

[S5] D. Wu, C. Wang, B. Huo, K. Hu, X. Mao et al., Photo- and electrocatalytic CO_2 reduction based on stable lead-free perovskite Cs2PdBr6. Energy Environ. Mater. (2022). https://doi.org/10.1002/eem2.12411

[S6] Y. Wang, C. Wang, Y. Wei, F. Wei, L. Kong et al., Efficient and selective electroreduction of CO_2 to HCOOH over bismuth-based bromide perovskites in acidic electrolytes. Chem. Eur. J. **28**, 2201832 (2022). https://doi.org/10.1002/chem.202201832

[S7] M.-N. Zhu, B.-W. Zhang, M.-R. Gao, P.-F. Sui, C. Xu et al., Electrochemically reconstructed perovskite with cooperative catalytic sites for CO2-to-formate conversion. Appl. Catal. B **306**, 121101 (2022). https://doi.org/10.1016/j.apcatb.2022.121101

[S8] R.P. Singh, P. Arora, S. Nellaiappan, C. Shivakumara, S. Irusta et al., Electrochemical insights into layered La_2CuO_4 perovskite: Active ionic copper for selective CO_2 electroreduction at low overpotential. Electrochim. Acta **326**, 134952 (2019). https://doi.org/10.1016/j.electacta.2019.134952

[S9] J.E. Pander, J.W.J. Lum, B.S. Yeo, The importance of morphology on the activity of lead cathodes for the reduction of carbon dioxide to formate. J. Mater. Chem. A **7**, 4093-4101 (2019). https://doi.org/10.1039/C8TA10752A

[S10] D. Wang, S. Dong, L. Wen, W. Yu, Z. He et al., Highly selective electrocatalytic reduction of CO_2 to HCOOH over an in situ derived hydrocerussite thin film on a Pb substrate. Chemosphere **291**, 132889 (2022). https://doi.org/10.1016/j.chemosphere.2021.132889

[S11] S.Y. Choi, S.K. Jeong, H.J. Kim, I.-H. Baek, K.T. Park, Electrochemical reduction of carbon dioxide to formate on tin–lead alloys. ACS Sustain. Chem. Eng. **4**, 1311-1318 (2016). https://doi.org/10.1021/acssuschemeng.5b01336

[S12] L. Jia, M. Sun, J. Xu, X. Zhao, R. Zhou et al., Phase‐dependent electrocatalytic CO_2 reduction on Pd_3Bi nanocrystals. Angew. Chem. Int. Ed. **133**, 21909-21913 (2021). https://doi.org/10.1002/ange.202109288

[S13] B. Kumar, V. Atla, J.P. Brian, S. Kumari, T.Q. Nguyen et al., Reduced SnO_2 porous nanowires with a high density of grain boundaries as catalysts for efficient electrochemical CO_2-into-HCOOH conversion. Angew. Chem. Int. Ed. **56**, 3645-3649 (2017). https://doi.org/10.1002/anie.201612194

[S14] H. Zhong, Y. Qiu, T. Zhang, X. Li, H. Zhang et al., Bismuth nanodendrites as a high performance electrocatalyst for selective conversion of CO_2 to formate. J. Mater. Chem. A **4**, 13746-13753 (2016). https://doi.org/10.1039/C6TA06202D

[S15] W. Ma, S. Xie, X.-G. Zhang, F. Sun, J. Kang et al., Promoting electrocatalytic CO_2 reduction to formate via sulfur-boosting water activation on indium surfaces. Nat. Commun. **10**, 892 (2019). https://doi.org/10.1038/s41467-019-08805-x
